# Inhibition of lipopolysaccharide-induced inflammation via the protective role of T regulatory cells in the fetal liver in a late-pregnancy preterm mouse model

**DOI:** 10.6061/clinics/2020/e1665

**Published:** 2020-11-02

**Authors:** Muhammad Siddiq, Fan Wang, Mi Xiao, Xiao Jie Lin, Nazira Fatima, Sara Iqbal, Umar Iqbal, Xian-Hua Piao, Li Liu

**Affiliations:** I Department of Neonatology, The First Affiliated Hospital of Xi’an Jiaotong University College of Medicine, Xi’an, Shaanxi, China; II Department of Biotechnology, Mirpur University of Science & Technology Pakistan, Pakistan and Laboratory Animal Center Xian Jiaotong University Health Science Centre, Xian Shaanxi, China; III Akhtar Saeed Medical & Dental College, Akhtar Saeed Trust Hospital & Farooq Hospital Lahore, Akhtar Saeed Medical & Dental CollegeAkhtar Saeed Trust Hospital & Farooq Hospital LahorePakistanPakistan; IV The Division of Newborn Medicine, Boston Children’s Hospital of Harvard Medical School, Boston, Massachusetts, U.S.A

**Keywords:** Forkhead Family Transcription Factor-3, Heme Oxygenase-1, Interleukin-6, Toll-Like Receptor-4, Lipopolysaccharide, Liver

## Abstract

**OBJECTIVES:**

This study intended to explore the effect of T regulatory cells (Tregs) in the perinatal liver against LPS-induced inflammation in a preterm birth mouse model. Moreover, the role of adoptive Tregs on the inflammatory response induced by LPS was also studied.

**METHODS:**

Female BALB/C mice were injected intraperitoneally (IP) with LPS dissolved in normal saline solution at a dose of 50 µg/kg. Spleens from pregnant mice were used to obtain Tregs. The expression of Forkhead family transcription factor-3 (Foxp3), Interleukin-6 (IL-6), Toll-like receptor-4 (TLR-4), and Heme oxygenase-1 (HO-1) were assessed from fetal liver tissues by polymerase chain reaction and western blotting.

**RESULTS:**

LPS administered to mice induced an inflammatory response in the perinatal liver, and this inflammatory response was negatively regulated by Tregs in the experimental group. Maternal-fetal tolerance was maintained by Tregs. Transmission of Tregs was estimated in different experimental groups based on the mRNA expression of TLR-4, IL-6, HO-1, and Foxp3.

**CONCLUSIONS:**

After analysis of the experimental data, it was determined that Tregs exhibited regulatory potential against LPS-induced inflammatory response. Further, it was concluded that the transmission of Tregs improved the mother’s immune tolerance against LPS-induced inflammation in the fetal liver.

## INTRODUCTION

Lipopolysaccharides (LPS) are endotoxins present in gram-negative bacteria and are considered a major player in driving the pathogenesis of septic shock (1). Cytokines and various inflammatory mediators are produced as a result of the LPS-induced stimulation of inflammatory cells. Treatment with LPS significantly increased the interleukin 6 (IL-6) production in the liver; IL-6 is a central mediator during liver infection. IL-6 signaling in effector T cells is essential for overcoming regulatory T cell (Treg)-mediated suppression of inflammation *in vivo* (2). IL-6 cooperates with IL-1β to block the suppressive effect of Tregs on CD^+^T cells, at least in part by controlling their responsiveness to interleukin-2 (3).

Tregs have a strong ability to initiate immune responses (4). Tregs are a subset of CD_4_^+^ cells that sustain peripheral immune homeostasis by inhibiting a range of inappropriate immune responses and establishing the balance between immune tolerance and activation (5). Treg development is maintained by Foxp3, which is responsible for encoding a transcription factor that is genetically defective in autoimmune and inflammatory syndromes in mice and humans (6). Foxp3-expressing Tregs play an indispensable role in immunological self-tolerance and immune homeostasis (3). Tregs significantly contribute to generating active immune tolerance in the developing fetus during pregnancy (7). Maternal Treg expansion is required for sustaining pregnancy and creates naturally occurring holes in the host defense that confer prenatal infection susceptibility (7).

Fetal growth restriction and preterm delivery are associated with decreased numbers of circulating Tregs (8). In murine Tregs, Foxp3 is upregulated and required for Treg growth and function, and is also used as an intracellular indicator to recognize Tregs (9). Heme oxygenase-1 (HO-1) expression is induced by Foxp3 and involved in Foxp3-mediated immune responses (10).

Heme deprivation enzyme is an inducible isoform of HO-1. HO-1 expression is enhanced in the liver due to a low dose of LPS. HO-1 has major immunomodulatory and anti-inflammatory properties (11). For the generation and maintenance of Tregs, HO-1 is valuable. In many conditions, HO-1 plays a protective role via its anti-inflammatory, anti-proliferative, and anti-apoptotic actions (12). HO-1 is constitutively expressed in a subpopulation of T regulatory cells (CD_4_+CD_25_), and HO-1 levels increase even further following T cell stimulation (13).

IL-6 has pro-inflammatory properties and is produced by a variety of cells as a result of trauma and infection. Upregulation of IL-6 expression is provoked by pro-inflammatory cytokines and chemokines (14). Due to intrauterine inflammation, TLR-4 is upregulated in the fundal region of the uterus (15). TLR-4 is involved in LPS-induced preterm birth, but has no role in inflammation-induced fetal death. Therefore, the significant clinical problem of inflammation-induced preterm birth reveals the signal transduction pathways that are responsible for the inflammatory state (15). In this study, the effect of T regulatory cells against LPS-induced inflammation was assessed in the perinatal liver in a preterm birth mouse model. Moreover, the role of adoptive Tregs in the inflammatory response induced by LPS was also studied.

## MATERIALS AND METHODS

### Animals

C57BL/6 male mice were pair mated with same age (ten-week-old) BALB/c female mice from the Laboratory Animal Center at the First Affiliated Hospital, Xi’an Jiao Tong University. Three mice in each group were used for the experiments and housed at 24±1°C with a sequential light/dark cycle, relative humidity of 55±5%, and free access to diet and water. Day zero of gestation was considered when a vaginal plug appeared. In our university, all mice were housed at the mouse facilities. The Institutional Animal Care and use Committee and Ethics Committee approved all the animal procedures (NO: 2017-1246). The model mice were assigned to different groups as follows:

1. Intraperitoneal (IP) injection of LPS; Intravenous (IV) injection of Tregs;
2. IP injection of LPS; IV injection of phosphate-buffered saline (PBS);
3. IP injection of PBS (Control group); and
4. IP injection of LPS (LPS group).

The model mice were analyzed at 1 h, 6 h, and 12 h after each treatment, and after delivery. First, the mice were anesthetized using 10% chloral hydrate (350 mg/kg), and then, fetal livers were harvested for analysis.

### Treg isolation and generation of the preterm birth mouse model

CD4+CD25+ Foxp3+Tregs were isolated from the spleens of healthy pregnant mice on day 14 by using a CD4+CD25+Regulatory T cell isolation kit (Miltenyi Biotech GMbH, Bergisch Gladbach, Germany) and LD column (designed for stringent depletion of unwanted cells), two MS columns (Column for the positive selection of cells suitable for the depletion of strongly magnetically labeled cells), and magnetic beads in the MACS cell separation product (MACS, Miltenyi, Biotech, Germany). Cells were stained with CD4-FITC (Miltenyi). The scatter signal and propidium iodide fluorescence were measured to exclude dead cells and cell debris. Tregs had a purity level ranging from 90% to 96% in all experiments. The number of Tregs in 200 µL of PBS were first counted; then, the suspensions were diluted appropriately and injected intravenously into pregnant mice.

A mouse model of preterm birth was established as reported previously (16). The mice received IP injections of LPS (*Escherichia coli*, serotype O55:B5; Sigma L-2880, dissolved in saline solution) at a dose of 50 µg/kg or an equivalent volume of PBS at 2 pm and 5 pm on day 17 of pregnancy. At 1 h before the first LPS injection, 200 µL of the Treg suspension (2×10^5^ cells) or PBS was injected. For sampling, 10% chloral hydrate (350 mg/kg) was used to anesthetize the mice, and the livers from six mice were harvested and examined at 1 h, 6 h, and 12 h after the second LPS injection, and then stored at -80°C for the analysis of gene and protein expression.

### Real-time reverse transcription polymerase chain reaction (qRT-PCR)

A tissue RNA Prep kit (OMEGA BIO-TEK, INC., Norcross, GA) was used to extract RNA from liver tissues. PrimeScript_TM_ RT Master Mix (TaKaRa, Shiga, Japan) was utilized as per the guidelines of the manufacturer to perform reverse transcription. To evaluate the mRNA levels of HO-1, Foxp3, IL-6, TLR-4, qRT-PCR was performed using an ABI PRISM 7900 real-time PCR SYSTEM (Applied Biosystems, Foster City, CA) with the SYBR R Premix Ex Taq_TM_ kit (TaKaRa); β-actin served as the reference gene. The specific primers used for Foxp3, IL-6, HO-1, and TLR-4 are listed in (Table 1). The fractional cycle was indicated by the cycle threshold (Ct) at which the PCR product was first detected above a fixed threshold.

### Western blotting

Liver tissues were lysed in RIPA buffer (Heart Biological, Xi’an, China). The Bradford protein assay (Bio-Rad Laboratories, Hercules, CA) was used for the measurement of total proteins (50 µg for HO-1 and Foxp3, 100 µg for IL-6 and TLR-4), which were separated by sodium dodecyl sulfate-polyacrylamide gel electrophoresis under reducing conditions, and then transferred onto a nitrocellulose membrane (Table 2). The membranes were blocked using 5% non-fat dry milk in Tris-buffered saline.

### Statistical analysis

Descriptive statistical analysis was evaluated by calculating the means of three replicates and their standard deviations. GraphPad Prism 8 (Two-way ANOVA) was used for statistical analysis. Results were considered significant at *p*<0.05.

## RESULTS

### Foxp3 expression in fetal liver tissues

**Figure 1:**
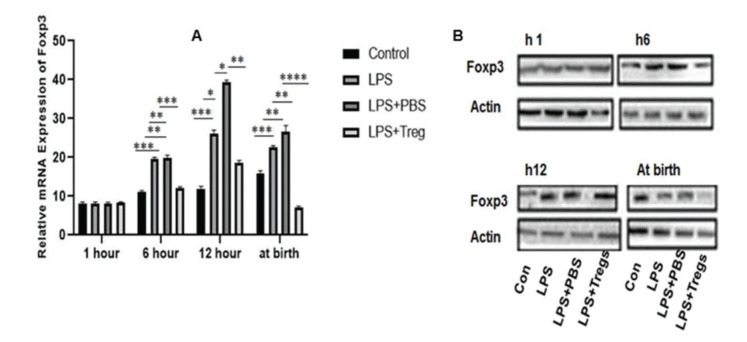
Statistical analysis of Foxp3 expression in different groups. (A) The expression levels of Foxp3 among different groups of model mice were analyzed. The mRNA expression of Foxp3 in the LPS and LPS+PBS groups was significantly higher than in the control group at 6 h, 12 h, and birth after the second administration of LPS (*p*<0.05). The mRNA expression of Foxp3 in the LPS+Tregs group was significantly lower than that in the LPS and LPS+PBS groups at 6 h, 12 h, and birth after second administration (*p*<0.05; *p<0.05, **p<0.01; ***p<0.001; ****p<0.0001). (B) The western blot results showed that the protein expression of Foxp3 in all groups followed a trend similar to that of the Foxp3 mRNA expression.

First, LPS (50 µg/kg) was administered to model mice 1 h before the injection of Tregs. No cases of death in either the mother or fetus were reported (Table 3). To evaluate the effect of Tregs on fetal hepatic tissues, the expression level of Foxp3 was measured following intrauterine infection. No significant difference in the Foxp3 protein and mRNA expression was observed among the groups after the first LPS administration, as shown in Figure 1. At 6 h, the Foxp3 expression levels among the groups were different. Adoptive Treg transfer markedly reduced the Foxp3 mRNA expression in the LPS+Tregs group at a different period; after 6 h, the Foxp3 expression level in the LPS+Tregs group presented a decreased expression compared to that in the LPS and LPS+PBS groups (**p<0.01). At 12 h, the Foxp3 expression level in the LPS+Tregs group differed significantly from that in the LPS and LPS+PBS groups (*p<0.03 and *p<0.05, respectively). In the LPS+Tregs group, the expression level of Foxp3 was decreased (*p*<0.01), compared to that in the control group. The expression of Foxp3 in the LPS+Tregs group was decreased (***p<0.001) at birth. After the second LPS administration, the expression level of Foxp3 was decreased specifically at birth, compared to that at the other indicated time points.

### IL-6 expression level in fetal liver tissues

**Figure 2:**
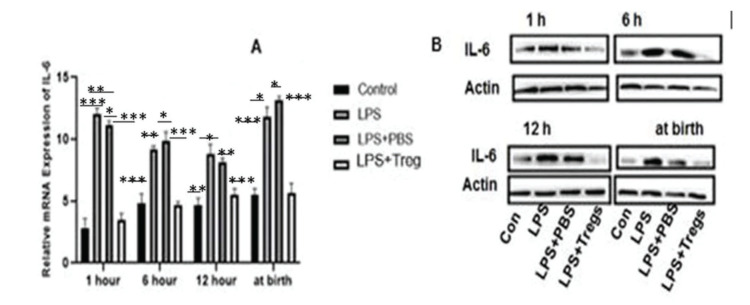
Expression of IL-6 in fetal liver tissues after maternal LPS administration. (A) The mRNA expression of IL-6 was significantly increased at the four indicated time points in the LPS+PBS group *versus* LPS group, compared to the levels in the PBS group. Adoptive transfer of Tregs markedly inhibited the LPS-induced upregulation of IL-6 mRNA expression (*p*<0.05, *p<0.05, **p<0.01, and ***p<0.001). (B) The western blotting results showed that the protein expression of IL-6 in the groups followed a trend similar to that of the IL-6 mRNA expression.

The protein and mRNA levels of IL-6 were measured in the fetal liver tissues at specified time intervals. However, the maternal transfer of Tregs minimized the effect of LPS on the IL-6 mRNA expression (LPS+Tregs group *versus* LPS group; 1 h, *p*=0.002; 6 h, *p*=0.01; 12 h, 0.001; and at birth, 0.0001). Maternal LPS administration markedly enhanced the mRNA expression of IL-6 in the fetal tissues at the specified time intervals (LPS+Tregs group *versus* control group: 1 h, *p*=0.003; 6 h, *p*=0.0001; *p*=0.001; *p*=0.0001). The protein expression of IL-6 following LPS stimulation was reduced after the transfer of maternal Tregs. These results suggested that maternal exposure to LPS increased the expression of other pro-inflammatory cytokines and IL-6 in the fetal liver. These elevated expression levels were considerably decreased following the adoptive transfer of Tregs. Likewise, the maternal transfer of Tregs significantly decreased the LPS-induced upregulation of the protein expression of IL-6. According to these data, the expression of IL-6 was increased in the fetal liver after maternal exposure to LPS. However, this increase in expression was diminished following the adoptive transfer of Tregs, as shown in Figure 2.

### HO-1 expression in fetal liver tissues

**Figure 3:**
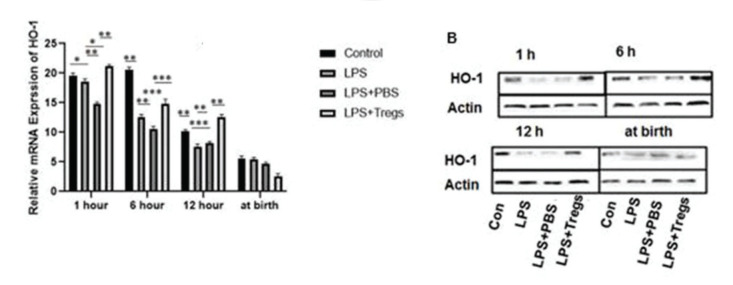
(A) In liver tissues, HO-1 expression after different treatments was measured via qRT-PCR. The differences were significant per the following criteria: *p<0.05, **p<0.01 and ***p<0.001. (B) HO-1 expression was decreased at birth in all groups, as examined by western blotting.

Fetal neonatal consequences and adverse pregnancy infection can be caused due to changes in effector immune system balance. In our experiments, there was a fluctuation in the HO-1 mRNA expression with time. The mRNA expression of HO-1 in the fetal tissues increased significantly following the maternal LPS administration at the specified time intervals (LPS group *versus* LPS+Tregs group; 1 h, *p<0.03; 6 h, **p<0.001; 12 h, *p<0.04) and decreased at birth. In PBS group (PBS group *versus* LPS+Tregs group at 1 h, **p<0.01; 6 h; ***p<0.001; 12 h *p<0.05), a fluctuation in the expression of HO-1 was observed, but at birth, there was no significant difference between the HO-1 expression levels among the groups. The HO-1 expression level decreased at birth (Figure 3).

### TLR-4 expression in fetal liver tissues

**Figure 4:**
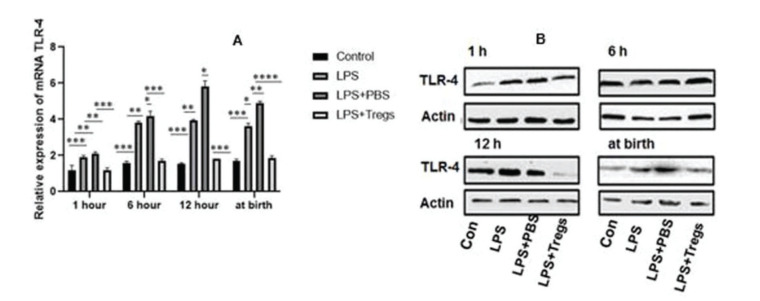
(A) After different treatments in liver tissues, the TLR-4 was statistically measured and examined by qRT-PCR. The significant difference among groups were *p<0.05, **p<0.01 and ***p<0.001. (B) In different time levels, mRNA expression was decreased after the transmission of Treg cells by western blot.

Altered expression of neonatal Toll-like receptors (TLRs) was involved in the heightening of infection and inflammation in term and preterm neonates; this is clinically evident in sepsis and is often associated with an increased rate of inflammatory disorders. TLR-4 is an important component of hepatic inflammation. The expression levels of TLR-4 in the different groups are shown in Figure 4. The administration of maternal LPS significantly induced the upregulation of the mRNA and protein expression of TLR-4 in fetal liver tissues, and upregulation of TLR-4 expression was almost completely abrogated by the adoptive transfer of Tregs.

## DISCUSSION

It has been reported that Tregs mediate the active immune response from the time of embryo implantation to protect the conceptus from maternal immune attack (17). Tregs are important mediators of the maternal immune adaptation needed for embryo implantation (18). The most important cause of preterm birth in developing as well as in developed countries is neonatal morbidity and mortality. Intrauterine infection is related to 40% of preterm births (19). Inflammation has been implicated as a mechanism responsible for preterm and term parturition. Inflammation is responsible for the recruitment of cells and molecules for suppressing infection (20).

These cells are fundamental to mediating maternal tolerance to the allogenic fetus in the grafting period during the premature stages of pregnancy. Tregs are not essential for the maintenance of late-stage allogenic pregnancy (21). Tregs can regulate immune cell responses directly at the fetal-maternal interface either by interacting with other cells or by inducing the expression of immune regulatory molecules (22).

In this study, it was experimentally shown that the expression of Foxp3 was significantly decreased in the LPS group, compared to that in the control group. In the LPS+Tregs group, the expression levels at different time points, *e.g.*, 1 h, 6 h, 12 h, and at birth, were restored, compared to that in the LPS group. With the passage of time, the expression of Foxp3 increased by the 12^th^ hour after the first LPS injection. Following the second LPS injection after birth, the Foxp3 expression decreased, compared to that at the previous time points (Fig 1A & B). IL-6 is a pleiotropic cytokine that has both pro- and anti-inflammatory properties. It plays a crucial role in the inhibition of autoimmune tissue inflammation (23). The protein expression level of IL-6 at different time intervals was assessed. The expression of IL-6 in the LPS+Tregs group at 1 h, 6 h, 12 h, and at birth decreased after the second LPS administration. The statistical analyses of the data of this study reveal that T regulatory cell therapy reduces the inflammatory action in the fetal liver by suppressing IL-6 expression (Fig 2A & B).

Tissue repair is enhanced by HO-1 (24), a major enzyme for prevention of inflammation. During late gestational inflammation, dysregulation in maternal immune response occurs and is mediated by HO-1 (25). The expression of HO-1 was examined in different organs following the inflammatory response, but the results regarding its expression in the liver are inconclusive (26). HO-1 is involved in Foxp3-mediated immune suppression (10). The mRNA expression level of HO-1 in the LPS+Tregs group at 1 h, 6 h, and 12 h increased, but HO-1 was downregulated at birth after the second administration of LPS (Fig 3A & B). The association between Tregs and upregulation of HO-1 expression has been rarely studied, but it has been concluded that HO-1, with the help of Tregs, upregulated the expression of IL-10, thus exerting anti-inflammatory effects (25). In another study, it was discussed that for the activation and proliferation of T regulatory cells, HO-1 played a vital role; HO-1 has also been reported to mediate the activity of CD^+^T cells via another mechanism (26).

Innate immune reactions in the host are induced by TLRs, including other components of the pro-inflammatory cascade, which involves prostaglandins, cytokines, chemokines, and other effector molecules; this cascade results in uterine contractions and rupture of the fetal membrane, which in turn, leads to labor (27). Studies have reported that LPS administration in allogenic mice causes multi-organ collapse (28). In LPS-induced preterm birth, TLRs play a vital role, but showed no effects in inflammation-induced fetal death (15). TLR-associated cytokine secretion depends upon gestational age (29). In this study, we evaluated the expression levels of HO-1, Foxp3, IL-6, and TLR-4 in the mice following different treatments. Inflammation was induced by LPS injection. Expression levels of these genes (TLR-4, IL-6, HO-1 and Foxp3) were increased under inflammatory conditions. The inflammatory conditions were overcome by Tregs within the time intervals analyzed in our late-pregnancy preterm mouse model. Our data revealed that the adoptive transfer of Tregs could suppress the inflammatory response in the liver of mice subjected to LPS-induced preterm birth, which was achieved by mediating the expression of IL-6, HO-1, Foxp3, and TLR-4 in the liver. During our study, it was assumed that Tregs possess the ability to move across the placenta; Tregs showed their potential to suppress the inflammatory action of LPS in the fetal liver. These cells presented a balanced immune response and are indicated as helpful in autoimmune disorders as well as for preventing allograft rejection.

## CONCLUSIONS

Tregs in the fetal immune system expressed their potential to exert protective effects against LPS-induced inflammatory response in a maternal mouse model. Further, it was also concluded that the transmission of T regulatory cells could improve maternal immune tolerance against LPS-induced inflammation in the fetal liver.

## AUTHOR CONTRIBUTIONS

Siddiq M and Liu L contributed for the conceptualization. Siddiq M, Fatima N, Xiao M, Iqbal S, Wang F, Piao XH and Lin XJ contributed with methodology. Iqbal U contributed with software. Iqbal U contributed with supervision. Liu L contributed with visualization. Siddiq M, Fatima N, Xiao M and Iqbal S contributed with writing � original draft. Siddiq M contributed with writing – review.

## Figures and Tables

**Table 1:** For qRT-PCR, the following primers were used.

Gene	Sequences (5′-3′)	PCR product size
Foxp3	Forward Primer 5′-AGGGCCAGCATAGGTGCAGG-3′ Reverse Primer 5′-AGTGCCTGTGTCCTCAATGGTC-3′	86 bp
HO-1	Forward Primer 5′-CTGGAGATGACACCTGAGGTCAA-3′ Reverse Primer 5′-CTGACGAAGTGACGCCATCTG-3′	150 bp
IL-6	Forward Primer 5′-CAACGATGATGCACTTGCAGA-3′ Reverse Primer 5′-CTCCAGGTAGCTATGGTACTCCACA-3′	142 bp
TLR-4	Forward Primer 5′-CATGGATCAGAAACTCAGCAAAGTC-3′ Reverse Primer 5′-CATGCCATGCCTTGTCTTCA-3′	179 bp
Actin	Forward Primer 5′-CATCCGTAAAGACCTATCTGCCAA-3′ Reverse Primer 5′-ATGGAGCCACCGATCCACA-3′	171 bp

**Table 2:** Antibodies used for western blotting.

Antibody	Host species	Dilution	Specificity	Manufacturer
Foxp3	Mouse	1:1000	Liver tissues	CST
HO-1	Mouse	1:200	Liver tissues	SNATA
IL-6	Mouse	1:200	Liver tissues	SNATA
TLR-4	Mouse	1:200	Liver tissues	SNATA

**Table 3:** Response of Tregs in LPS-induced mouse model.

No of groups	Number of mice delivered					Rate of preterm delivery	Delivery(Mean)
	Preterm			Term			
	n	Day 17	Day 18	Day 19	Day 20		
Control	6	0	0	1	5	0%	19.8
LPS	6	0	6	0	0	100%	18.0
LPS+PBS	6	0	6	0	0	100%	18.0
LPS+Tregs	6	0	5	1	0	83%	18.1
